# Cross-Linking, Morphology, and Physico-Mechanical Properties of GTR/SBS Blends: Dicumyl Peroxide vs. Sulfur System

**DOI:** 10.3390/ma16072807

**Published:** 2023-03-31

**Authors:** Agata Rodak, Agnieszka Susik, Daria Kowalkowska-Zedler, Łukasz Zedler, Krzysztof Formela

**Affiliations:** 1 Department of Polymer Technology, Faculty of Chemistry, Gdańsk University of Technology, Gabriela Narutowicza 11/12, 80-233 Gdańsk, Poland; 2 Advanced Materials Center, Gdańsk University of Technology, Gabriela Narutowicza 11/12, 80-233 Gdańsk, Poland; 3 Department of Inorganic Chemistry, Faculty of Chemistry, Gdańsk University of Technology, Gabriela Narutowicza 11/12, 80-233 Gdańsk, Poland; 4 Department of Molecular Biotechnology and Microbiology, Faculty of Chemistry, Gdańsk University of Technology, Gabriela Narutowicza 11/12, 80-233 Gdańsk, Poland

**Keywords:** ground tire rubber, SBS copolymers, blends, compatibility, recycling, melt blending

## Abstract

In this work, ground tire rubber and styrene–butadiene block copolymer (GTR/SBS) blends at the ratio of 50/50 wt%, with the application of four different SBS copolymer grades (linear and radial) and two types of cross-linking agent (a sulfur-based system and dicumyl peroxide), were prepared by melt compounding. The rheological and cross-linking behavior, physico-mechanical parameters (i.e., tensile properties, abrasion resistance, hardness, swelling degree, and density), thermal stability, and morphology of the prepared materials were characterized. The results showed that the selected SBS copolymers improved the processability of the GTR/SBS blends without any noticeable effects on their cross-linking behavior—which, in turn, was influenced by the type of cross-linking agent used. On the other hand, it was observed that the tensile strength, elongation at break, and abrasion resistance of the GTR/SBS blends cured with the sulfur system (6.1–8.4 MPa, 184–283%, and 235–303 mm^3^, respectively) were better than those cross-linked by dicumyl peroxide (4.0–7.8 MPa, 80–165%, and 351–414 mm^3^, respectively). Furthermore, it was found that the SBS copolymers improved the thermal stability of GTR, while the increasing viscosity of the used SBS copolymer also enhanced the interfacial adhesion between the GTR and SBS copolymers, as confirmed by microstructure evaluation.

## 1. Introduction

The increasing interaction and interdependence between countries (i.e., globalization processes) are the main causes of the growing consumerism [1], which reflects the unjustified acquisition of goods that do not meet needs resulting from social, individual, or environmental aspects. As one can imagine, this trend has critical implications for environmental protection, as it affects the increasing amounts of waste and influences the water and carbon footprint (i.e., an increase in demand corresponds to an increase in production). According to the World Bank, the main waste groups are food and green waste, glass, metals, waste paper, wood, plastic, rubber, and leather [2]. Although the first group is the largest (up to 44% of global waste), the challenge of polymer waste (i.e., plastic and rubber)—which has been on the agenda for many years—is no less significant. According to Geyer et al. [3], who conducted a detailed analysis of the production, use, and disposal of plastics, about 8300 million tons of plastics had been synthesized and released into the world by the time of publication. So far, five main streams of plastic waste management have been presented and used in practice: (i) landfilling, (ii) incineration and energy recovery, (iii) reduction, (iv) reuse, and (v) recycling.

The above examples and recycling problems mainly concern single-component plastics (with the possible presence of pigments, stabilizers, or catalysts in their structure). In the case of multicomponent polymer waste, it is much more difficult to take appropriate measures for effective and environmentally friendly management. Even more problematic are materials with reinforcements in their physical structure, especially those belonging to other material groups (e.g., metals, ceramics, and a wide range of organic and inorganic compounds). One of the most problematic materials, posing a significant threat to the environment and human life, is the tire—a sophisticated technical product that is used in almost every household.

Hitherto, a number of waste tire management approaches have been developed, which can be classified as civil engineering [4,5,6], steelmaking [7,8], pyrolysis/gasification/energy recovery [9], and reclaiming/devulcanization [10,11,12]. This variety of methods is due to the fact that, unlike thermoplastics—which can be reprocessed to obtain a value-added product—it is not possible to reverse the cross-linked structure of vulcanized rubber to obtain a raw material from which a fully vulcanized product can be obtained. This situation makes ground tire rubber (GTR) less commercially attractive, finding far fewer practical uses as a waste product. Due to the enormous amount of rubber waste generated, it is necessary to look for feasible and cost-effective solutions for the partial disposal of used tires. One such direct and simple solution appears to be the production of polymer blends and composites modified with scrap rubber [13,14,15].

The incorporation of modified or unmodified GTR into polymer systems is a topic that has been addressed and studied for many years. GTR is usually added to thermoplastics such as polyethylene (PE) [16,17,18], polypropylene (PP) [19,20,21], polyvinyl chloride (PVC) [22], etc. It is also common to create blends based on natural or synthetic rubbers [23,24,25].

This strategy is often aimed at obtaining thermoplastic elastomers (TPEs) that have the properties of rubber and the processability of thermoplastics. However, the GTR particles are incompatible with most polymer matrices due to their cross-linked structure (limited chain mobility), which limits the use of high GTR concentrations (above 50 wt%) [26,27]. To improve the interactions between the GTR and polymer phases, three main strategies can be considered: (i) devulcanization and/or surface modification, (ii) addition of elastomers and/or grafted polymers as modifiers, and (iii) dynamic or co-cross-linking [28]. Since the designed methods of rubber waste management should be as cheap and feasible as possible, from a practical point of view, the best way would be to find a suitable GTR/polymer system that lead to a product with new, satisfactory properties through the vulcanization process. Therefore, a good solution seems to be the use of a polymer that can be vulcanized using commercially available and applicable curing systems, and that can potentially create co-cross-links between the components.

Given the above statement, sulfur and peroxide systems—typically used in the rubber processing industry—are the best choices. Both, as shown in other studies [29,30,31], lead to effective revulcanization and/or co-cross-linking of GTR and other polymer matrices. However, in order to maximize the efficiency of the interaction between the phases as well as possible, it is necessary to select a matrix that can effectively utilize both the sulfur system and the peroxide system. Considering the above requirements, styrene–butadiene–styrene block (SBS) copolymers—which can be used as compatibilizers of polymer/waste rubber composites [32,33,34,35]—seem to be a very promising choice for development.

In the literature, the most popular application of GTR/SBS systems is asphalt modification [36,37,38,39]. Such a solution combines the properties of SBS (i.e., improved high-temperature performance and reduced temperature susceptibility) with the presence of GTR, reducing the price of the asphalt modifier. The results show that the overall performance of asphalts modified by GTR/SBS systems is better compared to asphalts modified separately by SBS or GTR [40].

However, to the best of our knowledge, the publications on waste-rubber-based composites using SBS copolymers as the matrix or modifier are rather limited. Recently, Stelescu et al. [41] investigated the structure and properties of materials based on vulcanized rubber waste and styrene–butadiene–styrene thermoplastic elastomer. The results showed that the addition of vulcanized rubber powder led to improvements in the physico-mechanical properties of SBS-based materials between the two polymer phases, which was explained by the similar structures of the SBS and styrene–butadiene rubber present in the vulcanized rubber powder. It is worth mentioning that styrene–butadiene rubber is also the main component of GTR.

Moreover, the data from the literature show that the use of thermoplastic modifiers might stabilize the thermo-mechanical treatment of cross-linked rubbers while also further processing waste-tire-rubber-based materials characterized by high GTR contents [27], representing a crucial step forward for the implementation of circular economy strategies.

The presented trends confirm the necessity of the development of GTR/SBS blends with well-defined composition and fully characterized processing and physico-mechanical properties. This approach allows for the tailoring of the performance properties of modified and highly modified asphalts, as well as for a better understanding of the interactions between GTR/SBS-based systems and asphalts.

Therefore, in this work, the processing, physico-mechanical, thermal, and morphological properties of 50/50 wt% GTR/SBS blends were characterized as a function of the SBS copolymer type (linear/radial) and cross-linking system (sulfur-based and peroxide-based). The experimental data presented in this study provide useful information about the interfacial interactions between the GTR and SBS phases, allowing for tailoring of the final processing and performance properties of GTR/SBS blends.

## 2. Materials and Methods

### 2.1. Materials

Ground tire rubber (GTR) obtained from passenger cars and truck tires, with a particle size of up to 0.6 mm, was supplied by Grupa Recykl S.A. (Śrem, Poland). The basic components of GTR are natural rubber (NR), styrene–butadiene rubber (SBR), butadiene rubber (BR), additives (curing system, activators, plasticizers, etc.), carbon black, silica, and ash. The composition of GTR, determined by thermogravimetric analysis, included rubbers and additives (63.1 wt%), and carbon black and ash content (36.9 wt%).

Four types of styrene–butadiene–styrene copolymers were selected and obtained from the Sibur Company (Moscow, Russia). The physico-mechanical properties of the selected components are presented in Table 1.

### 2.2. Sample Preparation

GTR was melt-blended with four different, commercially available types of SBS at a ratio of 50/50. The GTR/SBS blends were prepared in a Brabender^®^ internal mixer (type GMF 106/2 fromBrabender GmbH & Co. KG, Duisburg, Germany). The mixing temperature and time were 200 °C and 8 min, respectively, while the mixing speed was set to 60 rpm. After cooling the material for at least 24 h, the GTR/SBS blends were mixed with a suitable curing system: a sulfur-based system (composition in phr: stearic acid—0.3; zinc oxide—2.5; 2-mercaptobenzothiazole (MBT)—0.9; N-tert-butyl-benzothiazole sulfonamide (TBBS)—0.9; sulfur—1.5) or dicumyl peroxide (DCP, 2 phr), using laboratory two-roll mills with working space of 200 × 400 mm manufactured by Buzuluk Komarov (Komárov, Czech Republic). Two systems were used to evaluate the cross-linking behavior of the GTR/SBS blends pressed at 170 °C into 2 mm thick tiles using a PH-90 hydraulic press manufactured by ZUP Nysa (Nysa, Poland), with the optimal curing time determined according to the ISO 6502 standard.

### 2.3. Methodology

The Mooney viscosity of the rubber compounds was measured at 100 °C using an MV2000 Mooney Viscometer (Alpha Technologies, Akron, OH, USA) according to ISO 289-1.

The vulcanization process was studied and recorded using an Alpha Technologies Premier RPA (Hudson, OH, USA) according to the ISO 6502 standard. Further calculation of the cure rate index (CRI) was performed to determine the characteristic curing curve. This parameter is related to the cross-linking rate, giving insight into the differences between samples. The parameter was calculated on the basis of equations published in previous works [42,43].

FTIR analysis was performed in the range of 4000–650 cm^−1^ using a Momentμm microscope attached to a Nicolet iS50 FTIR spectrometer (Waltham, MA, USA) equipped with the Specac Quest single-reflection diamond attenuated total reflectance accessory.

The tensile strength and elongation at break were measured in accordance with the ISO 37 standard. Tensile tests were carried out on a Zwick Z020 machine (Ulm, Germany) at a constant speed of 200 mm/min. The results reported are an average of five measurements for each sample. Shore A hardness was assessed using a Zwick 3130 durometer (Ulm, Germany) in accordance with ISO 7619-1.

The swelling degree of the blends (approx. 0.2 g per sample) as a function of time was evaluated using equilibrium swelling in toluene (at room temperature). The swelling degree was calculated according to Equation (1):

(1) $$Q=\frac{m_{t}-m_{0}}{m_{0}}\times 100\%$$

where *Q* is the swelling degree (%), *m_t_* is the mass of the swollen sample after time *t* (g), and *m*_0_ is the initial mass of the sample (g).

The sol fraction was determined based on the difference in mass between the initial sample and the dried sample after extraction, according to Formula (2):

(2) $$F_{sol}=\frac{m_{0}-m_{k}}{m_{0}}\times 100\%$$

where *F_sol_* is the content of the sol fraction (%), *F_gel_* is the content of the gel fraction (%), *m*_0_ is the initial mass of the sample (g), and *m_k_* is the mass of the dried sample after extraction (g).

The density was determined by the Archimedes method as described in ISO 1183. All measurements were performed at room temperature in a methanol medium, without exception.

Abrasion resistance (ΔV_rel_) was measured according to the ISO 4649 standard by using a rotating cylindrical drum device from Gibitre Instruments (Bergamo, Italy). Before the measurement, the sample was weighed and then abraded over an abrasive test paper of grade 60 at a constant force of 10 N. After the distance of 40 m, the loss of the sample’s weight was determined. The abrasion resistance for the studied GTR/SBS blends was calculated using Equation (3):

(3) $${\Delta V}_{rel}=\frac{\Delta m_{t}\times \Delta m_{const}}{\rho_{t}\times \Delta m_{r}}$$

where ΔV_rel_ is the abrasion resistance (mm^3^), Δ*m*_t_ is the mass loss of the GTR/SBS blend (mg), Δ*m*_const_ is the defined value of the mass loss for the reference compound (No. 1 was used) (mg); *ρ*_t_ is the density of the GTR/SBS blend (g/cm^3^), and Δ*m*_r_ is the mass loss of the reference compound (mg).

The morphology of the GTR/SBS blends was characterized using a JEOL 5610 scanning electron microscope (Tokyo, Japan). Prior to analysis, the samples were coated with a thin layer of gold.

Thermogravimetric analysis (TGA) was performed using a Netzsch TG 209 apparatus (Selb, Germany). The mass of the samples was in the range of 10–12 mg, to ensure that the thermal treatment was performed homogeneously. The samples were tested in the temperature range of 35–800 °C and under a nitrogen atmosphere, at a heating rate of 10 °C/min.

## 3. Results and Discussion

### 3.1. Mooney Viscosity and Curing Characteristics

**Figure 1:**
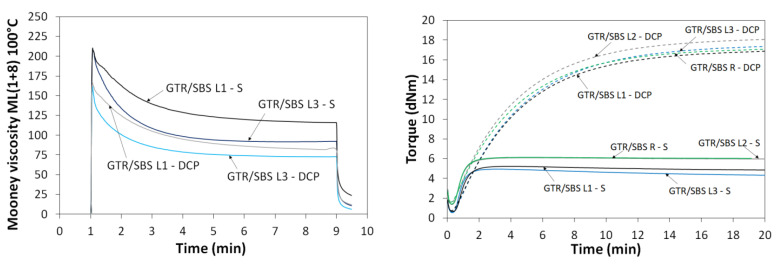
Mooney viscosity and curing characteristics (performed at 170 °C) of the studied GTR/SBS blends.

In order to investigate the processing of the studied GTR/SBS blends, Mooney viscosity measurements were performed at 100 °C, and the obtained results are presented in Figure 1. It was found that the Mooney viscosity could be determined only for GTR with SBS L1 and SBS L3, while for SBS L2 and SBS R the maximum initial value was reached. This is related to the melt flow behavior of SBS grades used; for SBS L2 and SBS R, the melt flow index was not specified by the manufacturer. As expected, GTR blended with SBS L3, with MFI_190 °C/5 kg_ = 16–25 g/10 min, was characterized by a lower Mooney viscosity than the GTR/SBS L1 blend (SBS L1 MFI_190 °C/5 kg_ = 3–9 g/10 min). It was also observed that, regardless of the SBS grade, samples with DCP showed lower Mooney viscosity compared to samples with the sulfur-based system. This was due to the melting temperature of DCP, which is ~40 °C [44,45]. As a result, melted DCP can act like a plasticizer during Mooney viscosity measurements at 100 °C, because it does not react at this temperature. As presented in Table 2, the Mooney viscosity at ML (1+4) 100 °C of the GTR/SBS L1 and L3 blends was in the range of 78.9–127.4 MU, while the Mooney viscosity at ML (1+8) 100 °C was slightly lower, at 73.0–116.5 MU, which was related to the prolonged heating of the material in the measurement chamber. For comparison, the Mooney viscosity at ML (1+4) 100 °C for commercial reclaimed rubbers can range from 65 to about 90 MU [46], which indicates that selected studied materials fit within this range.

Prior to formulating the studied materials into the desired shapes, the curing characteristics of the GTR/SBS samples were determined, and the results obtained are shown in Figure 1 and summarized in Table 2. As can be seen, changing the curing system had a significant effect on the cross-linking curves measured at 170 °C.

The minimum torque (M_min_) is a parameter that provides the first information about the sample processing. The processing characteristics of the sample were better for samples with lower M_min_. In this case, the only factor affecting this value was the grade of SBS copolymer used, and the effect of the curing system was negligible. For the GTR/SBS L1 and GTR/SBS L3 samples, the value of the M_min_ parameter was in the range of 0.5–0.7 dNm, while for samples GTR/SBS L2 and GTR/SBS R it was in the range of 1.4–1.6 dNm. The differences between the samples were related to the melt flow index of the SBS copolymers used, and the trends observed for M_min_ corresponded to the Mooney viscosity of the studied GTR/SBS blends (see Figure 1).

Maximum torque (M_max_) and extent of cure (ΔM) are parameters related to the stiffness and cross-link density of a material [47,48], which vary considerably between different types of curing additives. Comparing the results, the GTR/SBS blends cured with the sulfur-based system were characterized by M_max_ in the range of 4.9–6.2 dNm and ΔM = 4.4–4.7 dNm, while the GTR/SBS blends cured with DCP showed M_max_ in the range of 16.9–18.1 dNm and ΔM = 15.5–16.8 dNm. These results indicate that under the conditions studied, the SBS grade itself does not influence the course of the cross-linking process of GTR/SBS blends.

It is obvious that the efficiency of the curing system is strongly correlated with the cross-linking temperature. It was found that, regardless of the type of curing system, the GTR/SBS blends showed very short scorch times, which were in the range of 0.4–0.7 min. Moreover, as can be observed, for the GTR/SBS blends cross-linked by the sulfur-based system, the optimal cure time was in the range of 1.4–1.7 min, while for the GTR/SBS blend cured with DCP the optimal cure time was much longer, in the range of 9.5–9.9 min. These results confirm that in the studied conditions, the impact of the SBS grade on the cross-linking behavior of GTR/SBS blends is negligible. Moreover, the cure rate index (CRI) values clearly show that the sulfur system was more dynamic than the DCP at the test temperature (170 °C), which was related to the residence time of the material at elevated temperatures and the decomposition characteristics of the free radical initiators used.

### 3.2. FTIR Analysis

**Figure 2:**
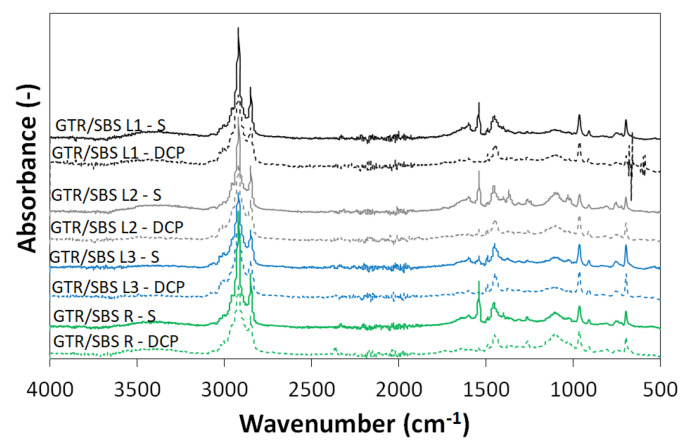
FTIR spectra of GTR/SBS blends as function of SBS grade and curing agent.

The FTIR spectra of the investigated samples are shown in Figure 2. The analysis of the obtained results shows that there were no significant differences between the samples. The bands of the C-H bonds of the CH_2_ groups present in the aliphatic chains of the elastomers are located at 2915 cm^−1^ and 2850 cm^−1^. The peak at about 1437 cm^−1^ is associated with C-H bonds of -C=CH_2_ groups, while the band at about 1367 cm^−1^ can be associated with C-H bonds of -CH_3_ groups. The band at 807 cm^−1^ corresponds to the skeletal vibration of the C-C bonds. In the range from 1100 cm^−1^ to 880 cm^−1^, C-O-C bonds as well as S=O, C-C, and C-O bonds can be found, which can be attributed to the structure of the components used and their transformation (oxidation of GTR, revulcanization, etc.). The only obvious difference between the spectra presented is the presence of an additional peak at 1540 cm^−1^ in samples with a sulfur system. This peak is related to zinc stearate, formed during the reaction between ZnO and stearic acid [49,50], which is not present in GTR/SBS cured with DCP.

**Figure 3:**
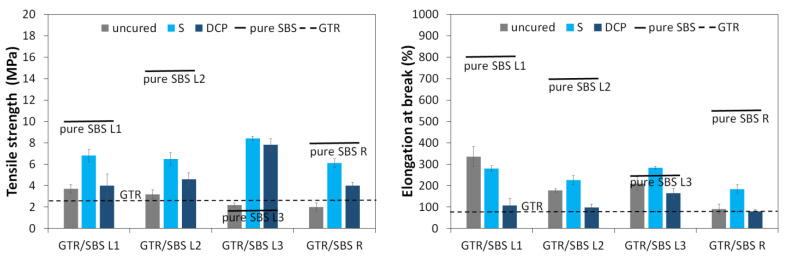
Tensile properties of GTR/SBS blends as function of SBS grade and curing system (data for GTR adapted from [52]; data for pure SBS copolymers were provided by the manufacturer).

As presented above, FTIR analysis indicated similar chemical structures of the prepared GTR/SBS blends, which simultaneously showed significant differences in rheological behavior and curing characteristics (see Table 3) or—as described in the next section—tensile properties (see Figure 3). This observation clearly shows that viscosity of the SBS copolymers (i.e., their molecular weight and polydispersity) is a very important parameter affecting the interfacial interactions between the GTR and SBS phases.

### 3.3. Physico-Mechanical Properties

The tensile properties of the studied GTR/SBS blends are presented in Figure 3. It was found that samples with DCP had lower tensile strength and elongation at break—by about 30% and 55%, respectively—compared to the specimens with sulfur. The tensile strength and elongation at break results obtained for the GTR/SBS blends were in the ranges of 6.1–8.4 MPa and 184–283% for samples cured with the sulfur-based system, respectively, and 4.0–7.8 MPa and 80–165% for samples cross-linked with DCP, respectively. This indicates that the sulfur-based system has a higher affinity for cross-linking of GTR/SBS blends than DCP. Poorer tensile properties compared to sulfur-cured products represent a common disadvantage of peroxide-cured vulcanizates [51].

Furthermore, it was observed that regardless of the cross-linking additives used, the highest tensile properties were determined for the GTR/SBS L3 sample, while the lowest tensile properties were determined for the GTR/SBS R sample. This is related to the flowability of the SBS copolymer (SBS L3 showed the highest melt flow index among the SBS copolymers used), which translates into better mixing efficiency between GTR and SBS.

To better understanding the effects of the cross-linking system on the tensile properties of the investigated system, uncured GTR/SBS blends were also investigated. Uncured GTR/SBS blends were characterized by tensile strength in the range of 2.0–3.7 MPa and elongation at break in the range of 91–336%—which, as expected, differed significantly from the cross-linked GTR/SBS blends. For comparison, the tensile strength and elongation at break of pure GTR are 2.6 MPa and 79%, respectively [52]. In addition, as predicted, most of the GTR/SBS blends were characterized by lower tensile strength than for pure SBS copolymers, with the exception of SBS L3 which, as mentioned above, may have been due to its having the highest melt flow index. More simply, the highest melt flow index indicates the lowest molecular weight of SBS L3 among the SBS copolymers used.

**Table 3:** Processing methods and tensile properties of thermoplastic composites modified with waste rubber and SBS copolymers described by different research groups.

Composition	Processing Method	Mechanical Properties		Observations	Reference
		Tensile Strength (MPa)	Elongation at Break (%)		
GTR/SBS 50/50 wt% with and without curing system	Internal mixer: 200 °C (8 min) Compression molding: at 170 °C (10 MPa, t_90_), and cold compression (5 min) for samples without the curing system	4.1–8.4 MPa (2.0–3.7 MPa without curing)	80–283% (91–336% without curing)	SBS with low viscosity enhanced processing and tensile properties due to the higher mixing efficiency between GTR and SBS	This study
HIPS/EVA/GTR 25/5/70 wt% compatibilized by SBS (up to 18 phr)	Internal mixer: 165 °C (8 min) Compression molding: at 180 °C (15 MPa, 10 min) and cold compression (8 min)	~6–8 MPa * (3.3 MPa for sample without SBS)	~115–245% (17.6% for sample without SBS)	SBS had a good compatibilizing effect and improved the tensile properties of the blends studied (the optimal SBS content was 12 phr)	[32]
HDPE/GTR 30/70 wt% compatibilized by SBS (up to 15 phr)	Internal mixer: 165 °C (8 min) Compression molding: at 165 °C (15 MPa, 9 min) and cold compression (8 min)	~12.3–14.8 MPa * (11.8 MPa for sample without SBS)	~240–260% (185% for sample without SBS)	SBS improved the mechanical properties and elasticity of the blends studied (the optimal SBS content was 12 phr)	[33]
SBS + 20 wt% waste rubber (footwear waste) with and without peroxide curing system	Internal mixer: 170 °C (7 min) Compression molding: at 170 °C (300 kN, 6 min) and at 45 °C (300 kN, 10 min)	~3.4–5.5 MPa * (~4.5 MPa for pure SBS)	~175–460% * (~580% for pure SBS)	The addition of vulcanized rubber powder (SBR-based) to SBS showed good compatibility between the two polymer phases, which was related to the similar structures of SBS and SBR. Dynamic cross-linking and grafting improved the mechanical properties of the studied materials. The investigated material showed good abrasion resistance.	[41]
LLDPE/GTR 34/66 wt% compatibilized by SBS and a DCP-based system (up to 10 wt%)	Kneading mixer: 185 °C (23 min) Compression molding: at 180 °C for 11 min (5 min preheating and 6 min of compression), and at room temperature for 4 min	~3.0–3.5 MPa * (3.1 MPa * for sample without SBS)	~50–113% * (43% * for sample without SBS)	Mechanical properties of the studied blends were improved by SBS (the optimal SBS content was 6 wt%)	[53]

* Estimated values based on available graphs.

Table 3 shows the summary with descriptions of the processing methods and tensile properties of thermoplastic composites modified with waste rubber and SBS copolymers prepared by different research groups. The data from the literature show that the mechanical properties of polymer/GTR blends modified with SBS copolymers are usually improved. This is due to the compatibilizing effect of SBS caused by proper encapsulation of cross-linked GTR [15] and possible co-cross-linking between the phases at the interfacial region [53].

As can be observed, the studied GTR/SBS blends at a ratio 50/50 wt% were characterized by tensile strength in the range of 4.1–8.4 MPa, which is higher than for SBS + 20 wt% waste rubber (footwear waste) (~3.4–5.5 MPa) [41], LLDPE/GTR 34/66 wt% compatibilized by SBS and a DCP-based system (up to 10 wt%) (~3.0–3.5 MPa) [53], and comparable to HIPS/EVA/GTR 25/5/70 wt% compatibilized by SBS (up to 18 phr) (~6–8 MPa) [32]. The tensile strength of the studied GTR/SBS blends was worse than for HDPE/GTR 30/70 wt% compatibilized by SBS (up to 15 phr) (~12.3–14.8 MPa) [33]; however, it should be noticed that in this case the reference sample without SBS possessed relatively high tensile strength (11.8 MPa).

The elongation at break of GTR/SBS blends at a ratio of 50/50 wt% was in the range of 80–283%, while for the thermoplastic composites modified with GTR and SBS copolymers described in the literature, the value of this parameter was in the range ~50–260%. For SBS + 20 wt% waste rubber composites, the elongation at break was in the range of ~175–460%, which was related to tensile parameters of the used thermoplastic elastomer and the composition of the waste rubber (footwear waste).

**Figure 4:**
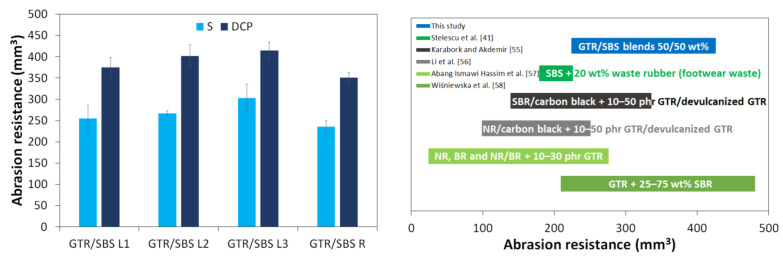
Abrasion resistance of GTR/SBS blends, and the comparison with the literature data [41,55,56,57,58].

Abrasion resistance is a very important parameter that determines the potential application of polymeric materials modified by waste rubbers in the production of footwear [41] or floor tiles [54]. The results of abrasion resistance for GTR/SBS blends are presented in the Figure 4. It was observed that regardless of the SBS grade, GTR/SBS blends cured using a sulfur-based system were characterized by higher abrasion resistance (ΔV_rel_ = 235–303 mm^3^) compared to the samples cured with DCP (ΔV_rel_ = 351–414 mm^3^). It was found that the GTR/SBS R blend was characterized by the best wear resistance, due to the radial structure of SBS R and having the highest viscosity among the SBS grades studied.

The literature data showed that the abrasion resistance of the prepared GTR/SBS blends at a ratio of 50/50 wt% was worse than for SBS + 20 wt% waste rubber (footwear waste), which was below 215 mm^3^ [41]. On the other hand, the studied 50/50 wt% GTR/SBS blends were characterized by abrasion resistance comparable to that of SBR/carbon black + 30–50 phr GTR/devulcanized GTR (~250–325 mm^3^) [55], NR/carbon black + 50 phr devulcanized GTR (~240 mm^3^) [56], NR + 30 phr GTR (266 mm^3^) [57], and GTR + 25–75 wt% SBR (219–470 mm^3^) [58]. As can be seen, in selected cases, the prepared GTR/SBS blends showed even better abrasion resistance considering the 50 wt% waste rubber content in the studied materials.

Table 4 presents a summary of the results for hardness, density, swelling degree, and sol fraction determined for the GTR/SBS blends. As can be observed, the hardness of the samples GTR/SBS L2 and GTR/SBS R was higher for samples cross-linked with dicumyl peroxide than when using the sulfur-based system, while in the case of the GTR/SBS L1 and GTR/SBS L3 samples the effect of the curing system on the hardness was negligible. This may be related to the more efficient mixing of the cross-linking system in GTR modified by SBS copolymers with higher flowability. It should be mentioned that the Mooney viscosity could be measured only for samples GTR/SBS L1 and GTR/SBS L3 (see Figure 1). The hardness of the studied materials was in the range of 66–78 Shore A and varied as a function of the SBS grade. Moreover, the hardness of the obtained materials was higher than that of pure GTR (57 Shore A). The highest hardness was determined for the GTR/SBS L3 sample, which also showed the highest tensile properties among the studied systems.

The results showed that the density of GTR/SBS blends is lower when the samples are cross-linked with DCP compared to the samples cured using a sulfur-based system, which is related to the density of the components used in the sulfur system (e.g., zinc oxide). However, this decrease in the density for samples with DCP is about 3%, so it is not a significant change. Moreover, the density of the GTR/SBS blends (in the range of 1.043–1.086 g/cm^3^) was lower than that of pure GTR (1.149 g/cm^3^).

The effects of the curing system and SBS grade on the swelling degree and the content of the sol fraction in the GTR/SBS blends were also analyzed. It was found that regardless of the grade of SBS copolymer used, the values of the swelling degree and sol fraction were lower for GTR/SBS blends cross-linked with DCP, indicating a more efficient cross-linking by this initiator.

### 3.4. SEM Analysis

**Figure 5:**
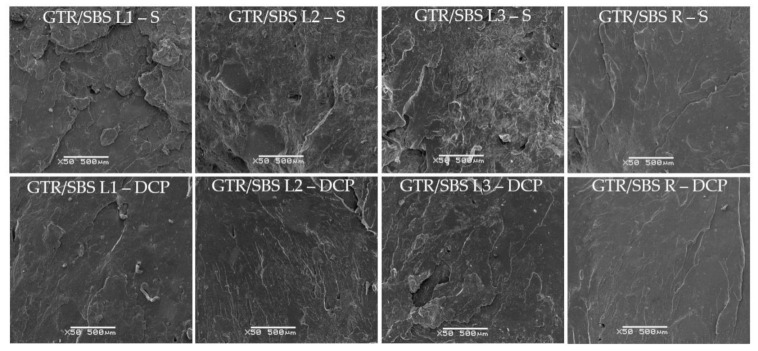
SEM images of GTR/SBS blends as function of SBS grade and curing system (magnification ×50).

Figure 5 shows the effects of the cross-linking system and the SBS copolymer grade used on the morphology of GTR/SBS blends. The SEM images show the surface perpendicular to the direction of loading, which was obtained by breaking the samples subjected to a static tensile test (the speed of the crosshead was 200 mm/min).

As can be observed, regardless of the type of SBS copolymer, samples cross-linked with DCP were characterized by a smoother surface compared to the samples cured with the sulfur-based system. Moreover, the ΔM and swelling degree values were higher for GTR/SBS blends cured with DCP compared to GTR/SBS blends cross-linked with the sulfur system (see Table 2 and Table 4). This indicates more efficient cross-linking of GTR/SBS blends by DCP, which could improve the compatibility between the GTR and thermoplastic phases [59].

Figure 5 shows that only for sample GTR/SBS R does the effect of the cross-linking agent on the surface of the samples after breaking seem to be negligible. This indicates that the radical structure of the SBS copolymer improves the interfacial adhesion between GTR and the SBS copolymer [15], which is also related to the high viscosity of the GTR/SBS R sample.

**Figure 6:**
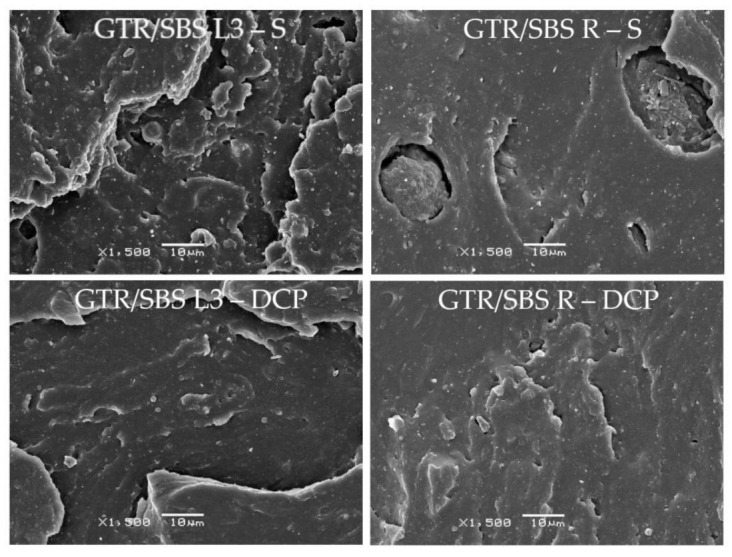
SEM images of GTR/SBS L1 (low-viscosity SBS) and GTR/SBS R (high-viscosity SBS) (magnification ×1500).

Considering data provided by the manufacturer of the used SBS copolymers, their viscosity increased in the order of SBS L3 < SBS L1 < SBS L2 < SBS R. Therefore, to better understand the effect of the SBS copolymers’ viscosity on the breaking mechanism, the surfaces of GTR/SBS R (SBS R with the highest viscosity) and GTR/SBS L3 (SBS L3 with the lowest viscosity) were investigated at ×1500 magnification, and the obtained SEM images are presented in Figure 6. The presented results clearly show that regardless of the cross-linking agent, the surface of the GTR/SBS R blend (with SBS R, characterized by high viscosity) is smoother and more homogeneous compared to that of the GTR/SBS L3 sample (with low-viscosity SBS L3).

### 3.5. Thermogravimetric Analysis

**Figure 7:**
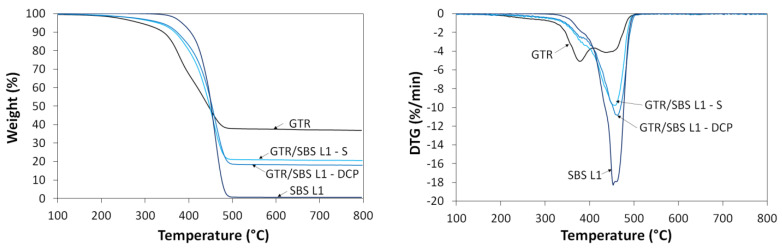
TGA and DTG curves for GTR/SBS blends.

The results of the thermogravimetric analysis of the studied GTR/SBS blends are shown in Figure 7 and summarized in Table 5. As can be seen in Figure 7, regardless of the curing agent used, the thermogravimetric (TGA) and derivative thermogravimetric (DTG) curves of GTR/SBS showed a similar trend, which fits between the curves of pure GTR and SBS. This indicates that blending GTR with SBS improves its thermal stability, which is related to the higher thermal stability of SBS compared to GTR. Figure 6 shows the results only for GTR/SBS L1 blends, but similar behavior was also observed for the other studied GTR/SBS blends, as can be estimated from Table 5, which indicates that the effect of the SBS grade on the thermal stability of the GTR/SBS blends was negligible.

Moreover, it can be observed that pure GTR is marked by two characteristic peaks on the DTG curve. T_max1_, at around 385 °C, corresponds to the presence of natural rubber and butadiene rubber, while T_max2_ at ~460 °C corresponds to the presence of styrene–butadiene rubber and butadiene rubber [60,61], which are the main rubber matrices used in the tire industry. The maximum thermal decomposition of SBS occurs at ~450 °C, which is due to the similar chemical structures of SBS and SBR.

The residual mass for GTR was 36.9%, which corresponds to the carbon black and ash content [62]. The presented results confirm that the waste rubber powder was produced from scrap tires. It was observed that in the case of GTR/SBS blends, the residual mass at 800 °C was slightly higher for GTR/SBS cross-linked with the sulfur-based system compared to the samples cured with DCP, which was related to the presence of ZnO used as an activator [63].

## 4. Conclusions

The aim of this study was to investigate the effects of the SBS copolymer grade and cross-linking agent on the Mooney viscosity, cross-linking characteristics, physico-mechanical properties, thermal stability, and microstructure of GTR/SBS blends, providing new insights into the interfacial interactions between GTR and SBS. The viscosity of the SBS copolymers increased in the following order: SBS L3 < SBS L1 < SBS L2 < SBS R. It was found that the GTR/SBS L1 and GTR/SBS L3 samples had the best processing parameters, and only for these materials could the Mooney viscosity be determined. The studied samples had Mooney viscosity at ML (1+4) 100 °C in the range of 78.9–127.4 MU, similar to the values determined for commercial reclaimed rubbers. The highest tensile strength was obtained for the GTR/SBS L3 sample, indicating that the application of SBS L3 copolymer—with the lowest viscosity among the SBS grades studied—allows for the formation of materials with the best processing and tensile properties, due to the higher mixing efficiency between GTR and SBS. On the other hand, considering the abrasion resistance of GTR/SBS blends, the best results were obtained with GTR/SBS R, due to the radial structure of SBS R and its high viscosity.

Studies on the effects of the cross-linking agent used—i.e., sulfur-based system vs. dicumyl peroxide (DCP)—showed that samples with DCP had lower Mooney viscosity values compared to GTR/SBS blends with a sulfur-based system. The results also confirmed that GTR/SBS blends cross-linked with a sulfur-based system had higher cure rates compared to samples cross-linked with DCP, which was related to the significant difference in the efficiency of the two initiators when used during cross-linking at 170 °C. The SEM images showed that the GTR/SBS cross-linked with DCP exhibited a smoother fracture surface compared to GTR/SBS cured with a sulfur-based system.

In conclusion, the investigated GTR/SBS blends showed relatively good tensile strength (4.1–8.4 MPa), elongation at break (80–283%), and hardness (66–78 Shore A), as well as abrasion resistance for selected blends (below 250 mm^3^). Considering the high GTR contents (50 wt%) in the studied GTR/SBS blends, along with the abovementioned mechanical properties, the prepared materials have a huge potential for the production of technical rubber goods, footwear, or floor tiles.

## Figures and Tables

**Table 1:** Physico-mechanical properties of the selected styrene–butadiene–styrene block copolymers.

Item *	Method	SBS Copolymer			
		SBS L1 (SBS L7322)	SBS L2 (SBS L7342)	SBS L3 (SBS L7417)	SBS R (SBS R7382)
Content of bound styrene (wt%)	Producer’s internal procedure	27.5–30.5	28.5–31.5	36.0–38.0	28.5–31.5
Melt flow index at 190 °C/5 kg (g/10 min)	Producer’s internal procedure	3.0–9.0	-	16.0–25.0	-
Tensile strength (MPa)	ASTM D 412	≥10.0	≥14.7	≥1.7	≥8
Modulus at 300% (MPa)	ASTM D 412	≥2.0	≥2.0	-	≥2.0
Elongation at break (%)	ASTM D 412	≥800	≥700	≥250	≥550
Hardness (Shore A)	ASTM D 2240	69–81	77–83	80–92	77–87
Volatile matter content (wt%)	ASTM D 5668	≤0.5			
Ash content (wt%): (a) Calcium stearate or zinc stearate (b) Silica	ASTM D 5667	≤0.3 ≤1.2			

* Data provided by producer in technical data sheet.

**Table 2:** Curing characteristics of the studied samples performed at 170 °C.

Properties	Sample Code							
	GTR/SBS L1		GTR/SBS L2		GTR/SBS L3		GTR/SBS R	
	S	DCP	S	DCP	S	DCP	S	DCP
Mooney viscosity ML (1+4) 100 °C	127.4	94.7	-	-	98.3	78.9	-	-
Mooney viscosity ML (1+8) 100 °C	116.5	82.5	-	-	91.8	73.0	-	-
Minimum torque (dNm)	0.7	0.7	1.4	1.6	0.5	0.6	1.4	1.6
Maximum torque (dNm)	5.2	16.9	6.2	17.1	4.9	17.4	6.1	18.1
Extent of cure (dNm)	4.6	16.2	4.7	15.5	4.4	16.8	4.7	16.5
Scorch time (min)	0.6	0.5	0.7	0.4	0.7	0.4	0.6	0.5
Optimal cure time (min)	1.7	9.7	1.6	9.6	1.4	9.9	1.6	9.5
Cure rate index (min^−1^)	90.9	10.9	111.1	10.9	142.9	10.5	100.0	11.1

**Table 4:** Hardness, density, swelling degree, and sol fraction of GTR/SBS blends.

Sample Code		Hardness (Shore A)	Density (g/cm^3^)	Swelling Degree (%)	Sol Fraction (%)
GTR *	-	57 ± 1	1.149 ± 0.007	169 ± 4	10.5 ± 0.3
GTR/SBS L1	S	66 ± 1	1.075 ± 0.008	318 ± 4	9.7 ± 0.2
	DCP	67 ± 1	1.043 ± 0.001	201 ± 2	7.1 ± 0.1
GTR/SBS L2	S	68 ± 2	1.072 ± 0.002	277 ± 6	9.4 ± 0.2
	DCP	73 ± 2	1.042 ± 0.001	186 ± 4	7.1 ± 0.2
GTR/SBS L3	S	78 ± 2	1.086 ± 0.004	368 ± 10	12.2 ± 1.1
	DCP	76 ± 2	1.044 ± 0.003	228 ± 2	9.2 ± 0.1
GTR/SBS R	S	67 ± 2	1.074 ± 0.008	248 ± 4	9.6 ± 0.1
	DCP	70 ± 2	1.043 ± 0.006	205 ± 3	9.0 ± 0.3

* Data for GTR with particle size 0.4 mm compressed at 180 °C, adopted from Ref. [52].

**Table 5:** Thermal decomposition temperatures and residue mass of GTR/SBS blends.

Sample Code		Decomposition Temperature (°C)				Residue Mass at 800 °C
		T_−2%_	T_−5%_	T_−10%_	T_−50%_	
GTR	-	230.9	290.0	342.4	445.3	36.9
SBS L1	-	365.7	384.2	404.2	451.5	0.7
GTR/SBS L1	S	263.4	330.4	368.6	448.1	20.6
	DCP	267.8	338.2	373.7	453.5	17.9
SBS L2	-	362.2	380.0	399.0	451.9	0.4
GTR/SBS L2	S	264.2	330.9	370.0	449.3	19.9
	DCP	264.3	336.9	373.7	453.1	18.0
SBS L3	-	353.4	376.7	395.3	454.0	0.2
GTR/SBS L3	S	262.7	330.7	370.2	449.6	19.8
	DCP	255.2	332.8	371.7	452.6	18.9
SBS R	-	363.4	381.2	399.7	452.5	0.8
GTR/SBS R	S	259.3	329.0	369.1	448.3	20.3
	DCP	269.6	340.6	376.6	454.3	18.3

## Data Availability

Not applicable.
